# Determinants of breast self-examination practice among women in Surabaya, Indonesia: an application of the health belief model

**DOI:** 10.1186/s12889-019-7951-2

**Published:** 2019-11-27

**Authors:** Triana Kesuma Dewi, Karlijn Massar, Robert A. C. Ruiter, Tino Leonardi

**Affiliations:** 1 Department of Clinical Psychology and Mental Health, Faculty of Psychology, Universitas Airlangga, Surabaya, Indonesia; 2 Department of Work and Social Psychology, Faculty of Psychology and Neuroscience, Maastricht University, Maastricht, Netherlands; 3 Department of Educational and Developmental Psychology, Faculty of Psychology, Universitas Airlangga, Surabaya, Indonesia

**Keywords:** Breast cancer, Breast self-examination, Champion’s health belief model, Cues to action, Indonesian women, Perceived benefits, Perceived barriers, Self-efficacy

## Abstract

**Background:**

Breast cancer has become one of the most common causes of mortality among Indonesian women. Many women in Indonesia present with late-stage breast cancer, negatively affecting prognosis and treatment outcomes. Better prognosis of breast cancer will be achieved if it is diagnosed in an earlier stage, thus efforts to detect breast cancer earlier are important. Breast Self-Examination (BSE) is considered as an important first step to encourage women to actively be responsible for their own health, especially for women in low-and middle-income countries with limited resources and access to other forms of preventive healthcare (e.g., screening programs). The present study aimed to predict BSE practice among women in Surabaya, Indonesia using the Health Belief Model (HBM).

**Methods:**

This investigation was a cross-sectional survey which was distributed among 1967 women aged 20–60 years. The Indonesian version of Champion’s Health Belief Model Scale (I-CHBMS) was used to explain self-reported BSE practice. Logistic regression analysis was used to examine the association of HBM variables with BSE practice.

**Results:**

44.4% of the respondents indicated they had performed BSE. Further, the results indicated that the HBM variables were significantly associated with BSE practice. Specifically, higher perceived benefits and self-efficacy, lower perceived barriers and less cues to action were unique correlates of BSE practice. The result also showed that perceived severity and susceptibility were not associated with BSE practice.

**Conclusion:**

This study indicated that several HBM constructs significantly associated with BSE practice among Indonesian women, suggesting that BSE health education programs should emphasize the perceived benefits of BSE, focus on increasing women’s self-efficacy to address and overcome perceived barriers in performing BSE, and help them in identifying personally relevant cues to action.

## Background

Globally, breast cancer is the most common type of cancer, especially in low-and middle- income countries [1]. For example, as of 2012, Indonesia had a breast cancer incidence rate of 40.3 per 100,000 population and a mortality rate of 16.6 per 100,000 [2]. Breast cancer accounted for 16.7% of all cancer cases (ranking first among types of cancer) and 11% of all deaths (ranking second after lung cancer) [3].

In Indonesia, most cancer patients are age 35 years or older; they come from diverse socio-economic backgrounds, but mostly live in urban areas [4]. The medical costs of managing, ranging from diagnosis to various treatments such as chemotherapy and radiotherapy, are high and are increasing every year [5, 6]. One factor that greatly increases the cost of cancer treatment is that most patients are already at an advanced stage of the disease when they first seek healthcare. Therefore, early cancer detection programs are important in Indonesia; even though early detection would not decrease the incidence of breast cancer, it would help improve the prognosis and treatment outcomes, which could ultimately reduce mortality rates [7], while also reducing the cost of treatments.

Regular breast self-examination (BSE), combined with breast self-awareness, is one of strategy aimed at achieving early breast cancer detection, especially in low- and middle-income countries where access to other early detection methods, such as mammography and ultrasonography, is limited. BSE is uniquely suited for populations with limited access to formal healthcare: it is inexpensive, non-invasive, simple to perform, and does not depend on a health practitioner’s assistance. Furthermore, performing regular BSE enhance breast awareness and encourage women to take more active responsibility for their own health [6, 8].

The Indonesian Ministry of Health launched its ‘National Movement for Prevention and Early Detection of Breast and Cervical Cancer’ program in 2015. This programme sought to encourage women age 20 and older to go to a primary healthcare center (PHC) for regular clinical breast examinations (CBEs). It is also provided BSE education and encouraged women to practice BSE at least monthly [5]. The effort to increase use of BSE as an early home-based screening for breast cancer constitutes a critical part of early detection activity.

Research in low-and middle-income countries has indicated that regular BSE is associated with the identification of breast cancer in an earlier stage, and thereby with reduced mortality [9]. However, early detection, whether through BSE or by other means, must be followed up with prompt diagnosis and effective treatment [10]. In low- and middle-income countries, as is the case in Indonesia where most cancers are diagnosed at a relatively late stage, BSE, followed up by CBE at a healthcare facility, could be effective in increasing early detection of breast cancer and in improving treatment outcomes [11].

Although exact numbers on BSE practice among Indonesian women are lacking, a study conducted among university students from low and middle income, and emerging economy countries indicated that BSE practice among Indonesian students was higher (64.5%) compared to other South East Asian countries, i.e., Laos, Philippines, Singapore and Thailand, where BSE practice ranged between 19.7–58.2% [12]. Moreover, studies in Muslim samples indicated that BSE practice among Saudi, Turkish, and Iranian women was 41.6, 39.5 and 41.9% respectively [13–15].

Several studies have shown positive associations between cancer screening behaviors (including BSE) and psychological variables such as self-efficacy and attitudes, and the Health Belief Model (HBM) in particular has proven to be a valid tool to predict such screening behaviors among women in low- and middle-income countries [15–17]. The HBM [18] explains health behavior as being determined by personal beliefs or perceptions about a disease, as well as by the strategies available to an individual to reduce the occurrence of the disease [19]. Specifically, according to this model, people’s beliefs and perceptions influence their health behavior, such that when individuals perceive that they are at risk for the disease they will perform the health behaviors necessary to prevent it, if they are reminded of these behaviors and expect anticipated positive health outcomes from performing them [18].

The most recent conceptualization of the HBM includes the following components [20]: perceived susceptibility, or one’s perceived personal vulnerability to the risk of incurring the health condition in question; perceived severity, or the degree of personal harm expected if one were to incur the health condition; perceived benefits, or the positive results expected from performing a certain health behavior; perceived barriers, meaning the perceived costs or negative attributes of performing a health behavior; cues to action, or the internal or external triggers that encourage the performance of the health behavior; and self- efficacy, or one’s confidence in his or her ability to perform the health behavior.

Champion [21] was the first to apply the HBM framework to investigating women’s beliefs regarding breast cancer and breast screening behavior, including BSE and mammography, resulting in the development of Champion’s Health Belief Model Scale (CHBMS) [22, 23]. This scale has been widely used to predict breast cancer screening rates, and has been adapted to many languages and cultural settings such as Iran [24], Taiwan [25], and Turkey [26]. The present study’s aim was to investigate whether the HBM variables are correlates of BSE practice among Indonesian women.

Previous research demonstrated the ability of HBM to predict a range of health-related behavior, including breast cancer screening practice [19]. Various results related to the significance and magnitude of HBM factors in predicting BSE practice have also been reported [27–29]. However, due to specific characteristics of the Indonesian population, further exploration of the relationship between HBM and BSE practice among Indonesian women is needed. This population is characterized by limited health care resources – for example, only 7.6% of all PHCs in Indonesia that provided early detection services for breast and cervical cancer [30] – and certain psycho-socio-cultural characteristics, such as fatalistic beliefs about health and illness, along with embarrassment about discussing or performing breast examinations. Knowledge about the determinants of BSE could inform health education programs designed to create breast cancer awareness and could stimulate regular BSE among Indonesian women.

## Methods

### Study design and participants

A cross-sectional study was conducted in Surabaya, the capital of East Java and the second largest city in Indonesia. Surabaya has 31 sub-districts, each of which comprises 3 to 8 villages. In the 2010 census, the total population of Surabaya was 2,599,796; women age 20 to 60 represented 30% of the population [31].

To obtain representative data, we randomly selected 76 villages of the total of 165 villages in Surabaya, including both rural and urban areas. Participants were recruited by trained research assistants, and the monthly meetings of PKK (‘Women’s Family Welfare Movement’, a nation-wide women’s NGO that seeks to reach every wife in a certain geographic area), were used to recruit female participants. Research assistants approached PKK members during these meeting and asked them to participated in the research. Most PKK members who attended these meetings agreed to be subjects. Specific inclusion criteria were: 1) being age 20–60 years old, 2) living in Surabaya, and 3) being literate in the Indonesian language. The research assistants explained the study purpose, which was also explained in the information sheet attached to the questionnaire. Some women took questionnaires home to distribute among their community members or to their daughters. The completed questionnaires were then collected by a PKK representative in each village. Completion time for the questionnaire was between 10 and 15 min. Care was taken to assure the confidentiality of the data obtained, and all participants provided written informed consent to participate in the study. A total of 2173 participants completed the questionnaire, but we had to exclude 206 participants due to incomplete responses. The final sample consisted of 1967 women. The data were collected between September 2016 and January 2017.

### Study instrument and data collection

After providing their informed consent, participants completed the CHBMS. We used the Indonesian version of the CHBMS (the I-CHBMS) to investigate the HBM variables; see also [32]. The scale is specifically targeted at breast cancer and BSE and consists of 42 items with 5-point response scales ranging from ‘strongly disagree’ (1) to ‘strongly agree’ (5). The I-CHBMS contains six subscales, align with the six item in the HBM: perceived susceptibility, six items, e.g., ‘I am very worried about getting breast cancer’; perceived severity, 12 items, e.g., ‘Having breast cancer will endanger my relationship’; perceived benefits, five items, e.g., ‘BSE can help me in finding a lump’; perceived barriers, eight items, e.g., ‘I am afraid I’m not able to do BSE’; cues to action, eight items e.g., ‘I often do things that can improve my health status’; and self-efficacy three items, e.g., ‘I was able to do my own BSE last month’. Total scores were calculated for the subscales of the I-CHBMS. All subscales had an acceptable to very good internal consistency reliability, with Cronbach’s alpha coefficients (respectively) of .84, .87, .82, .83, .81, .67. The item-total correlation ranged from *r* = .37 to *r* = .78.

After completing the I-CHBMS, participants answered some questions on socio-demographic characteristics, including age, level of education, and marital status. We also asked whether participants had experienced any breast affliction, whether they had a history of cancer in the family, and whether they had ever performed BSE. All these questions were answered either yes or no.

### Data analysis

The data were tabulated in excel worksheets and analyzed using IBM SPSS (version 22.0). The significance level was set at *p* < .05. Frequency distributions described the socio-demographic characteristics of the participants, and chi-square tests were used to compare demographic properties between women performing BSE (either regularly or irregularly) and those who did not perform it at all. Independent-sample t-test were employed to compare HBM constructs between BSE performers and non-performers. To examine the association between HBM variables and BSE practice among the participating women, logistic regression analysis was used.

## Results

After removal of incomplete response, the sample consisted of 1967 women from Surabaya, with a mean age of 36.17 (SD = 11.39). Most were married (72.3%), and more than half (53.4%) had graduated from high school. Of all participants, 44.4% had at performed BSE at least once. As shown in Table 1, BSE practice was associated with age, education, history of breast afflictions, and family history of cancer (*p*’s < .05).

**Table 1:** Socio-demographic characteristics of the participants

	BSE performers (*n* = 874)		BSE non-performers (*n* = 1093)		Statistics
	N	%	N	%	
Age					*χ*^*2*^ (1) =5.892, *p* < .001
≤ 40	536	61.30	728	66.66	
> 40	338	38.70	365	33.40	
Marital status					χ^2^ (2) = .729, *p* = .69
Single	197	22.50	264	24.20	
Married	639	73.10	784	71.70	
Widowed/divorced	38	4.30	45	4.10	
Education					χ^2^ (3) =102.342, *p* < .001
University	258	29.50	175	16	
High school	486	55.60	565	51.70	
Secondary	80	9.20	201	18.40	
Primary	50	5.70	152	13.90	
History of breast affliction					χ^2^ (1) =10.48, *p* < .001
Yes	34	3.90	17	1.60	
No	840	96.10	1076	98.40	
Family history of cancer					χ^2^ (1) =28.437, *p* < .001
Yes	168	19.20	117	10.70	
No	706	80.80	976	89.30	

Independent samples t-tests (see Table 2 for an overview) revealed that perceived benefits, cues to action, and self-efficacy were significantly higher among BSE performers than among women who never performed BSE (*t*’s > 6.48, *p*’s < .001). Additionally, the perceived barriers were significantly lower among BSE performers (*p* < .001). The groups did not differ with respect to perceived susceptibility and perceived severity of developing cancer (*t*’s < − 1.22, *ns*).

**Table 2:** Comparison of Health Belief Model factors among performers and non-performers of BSE

Variable	Performing BSE		*t*	df	*p*
	Yes	No			
	Mean (SD)	Mean (SD)			
Susceptibility	14.11(4.26)	14.11(4.05)	−.01	1965	.99
Severity	38.63(8.53)	39.10 (8.42)	−1.22	1965	.22
Benefits	20.27(2.67)	18.46 (3.23)	13.35	1963^*^	<.001
Barriers	16.34 (4.75)	19.52 (5.05)	−14.25	1915^*^	<.001
Cues to action	29.38 (4.72)	27.88 (5.40)	6.48	1950^*^	<.001
Self-efficacy	11.26 (2.06)	9.03 (2.56)	20.92	1965^*^	<.001

^*^Levene’s test indicated unequal variances (F’s > 14.12, *p’s* < .001) so degrees of freedom were adjusted

Lastly, logistic regression was used to explain the probability of BSE practice. First, we looked at the predictive value of the HBM components (Model 1). The results showed that the HBM variables were significantly associated with BSE practice (χ^2^ (6) = 485.92, *p* = .0001) and accounted for 29.3% of the variance of BSE practice. Next, we examined whether controlling for socio-demographic characteristics (age, marital status, education, history of breast affliction, and family history of cancer) would change the predictive value of the HBM components in explaining BSE practice, by including the socio-demographic variables in Model 2 in the first step, and the HBM variables in the second step. The results show that this model significantly predicted BSE practice (χ^2^ (11) = 553.35, *p* = .0001) and explained 32.8% of the variance in whether participants had ever utilized this screening method. There was 3.5% extra variance explained by sociodemographic variable in this model, compare to Model 1. Table 3 presents the full results.

**Table 3:** Hierarchical Logistic regression analysis of Health Belief Model factors for predicting BSE practice

Variables	Model 1^a^						Model 2^b^					
	B (S.E)	Wald	df	OR	OR 95% CI	*p*	B (S.E)	Wald	df	OR	OR 95% CI	*p*
Sociodemographic												
Age > 40 (ref = age ≤ 40)							.27 (.12)	5.44	1	1.32	1.05–1.65	.02
Married (ref = single, widow/divorced)							−.06 (.13)	.21	1	.95	.74–1.20	.65
University, high school (ref = primary, secondary school)							.90 (.14)	43.6	1	2.45	1.88–3.20	<.001
Having history of breast affliction (ref = no)							.60 (.34)	3.06	1	1.82	.93–3.56	.08
Having family with cancer (ref = no)							.49 (.15)	10.4	1	1.63	1.21–2.20	<.001
HBM constructs												
Susceptibility	.02(.01)	1.87	1	1.02	.99–1.04	.17	.01(.01)	.78	1	1.01	.99–1.04	.38
Severity	−.01 (.01)	.77	1	.99	.98–1.01	.38	.00 (.01)	.001	1	1	.99–1.01	.98
Benefits	.09 (.02)	20.28	1	1.1	1.05–1.14	<.001	.08 (.02)	16.2	1	1.09	1.04–1.13	<.001
Barriers	−.08 (.01)	42.36	1	.93	.91–.95	<.001	−.08 (.01)	40.10	1	.93	.90–.95	<.001
Cues to action	−.06 (.01)	22.31	1	.94	.92–.97	<.001	−.06 (.01)	26.31	1	.94	.91–.96	<.001
Self-efficacy	.36 (.03)	176.89	1	1.44	1.36–1.51	<.001	.36 (.03)	163.93	1	1.43	1.35–1.51	<.001

^a^Logistic regression with HBM constructs, Model χ^2^ (6) =485.92, *p* < .001, *R*^2^ = .293 (Nagelkerke)

^b^Logistic regression with sociodemographic characteristics + HBM constructs, Model χ^2^ (11) =553.35, *p* < .0001, *R*^2^ = .328 (Nagelkerke)

Unique correlates of BSE practice in Model 1 were perceived benefits, perceived barriers, cues to action, and self-efficacy (*p* ‘s < .001). The women who perceived greater benefits of BSE (OR = 1.1, CI = [1.05,1.14]), and indicated higher self-efficacy (OR = 1.44, CI = [1.36,1.51), but perceived fewer barriers (OR = .93, CI = [.91,.95]) and, surprisingly, had fewer cues to action (OR = .94, CI = [.92,.97]) were more likely to perform BSE. When socio-demographic variables were controlled in Model 2, the HBM components which associated with BSE practice were identical to those in the first model: perceived benefits, perceived barriers, cues to action and self-efficacy were among HBM components which explained BSE behavior. In addition, age (OR = 1.32, CI = [1.05,1.65]), education background (OR = 2.45, CI = [1.88,3.20]) and having family members with cancer (OR =,1.63 CI = [1.21,2.20]) were each associated with BSE practice, suggesting that women who were older, highly educated and had family members with cancer were more likely to have engage in BSE practice.

## Discussion

Breast cancer is the most predominant cancer among Indonesian women. Early detection and treatment of the disease have been shown to decrease mortality rates [33], but in Indonesia, almost 70% of breast cancer patients present in at a late stage of the disease, negatively affecting survival rates [34, 35]. Therefore, early detection strategies are important, and BSE is one of the best strategies applicable to achieve both breast (cancer) awareness and earlier detection of breast problems. Especially in low-and middle-income countries where health-related resources are limited, and where socio-cultural influences tend to make women more hesitant to discuss issues related to breast health, empowering women to examine their own breasts is an important first step [36]. The aim of the current research was therefore to investigate which psychosocial variables affect the likelihood of women’s performing of BSE.

The findings of the present study seem promising, since 44.4% of the participants indicated they had previously performed BSE. Moreover, an older age, higher education, and having a history of family member with cancer were all positively correlated with performing BSE in this sample. These results support the findings of previous studies that older and well-educated women were more likely to perform breast cancer screening behavior [14, 29], possibly because of increased knowledge and awareness.

In line with the HBM [18], perceived benefits, perceived barriers, cues to action, and self-efficacy all impacted the likelihood that a woman would perform BSE. In contrast to expectation, however, the regression analysis revealed a *negative* association between cues to action and the likelihood of performing BSE. This result might be due to how this variable was measured: Cues to action were probed by asking about general positive health behaviors, such as maintaining a healthy diet, adherence to physician recommendations, regular exercise, and an ability to find health information, rather than asking about internal or external triggers that encourage BSE performance. We speculate that the participants who indicated that they had more of those type of cues to action might also have had higher optimism regarding their health status. Although in most cases optimism about one’s health is associated with greater psychological and physical well-being, some studies have revealed less desirable outcomes, such as reduced health protective behavior [37, 38]. In some cases, this health optimism may thus be unrealistic, including perceptions of being invulnerable to the disease, especially since these women already feel that they are taking good care of their health. Additionally, people with positive general health behaviors – measured here by the cues to action subscale of the HBM – tend to think that negative events are less likely to happen to them than to other people [39], which might cause them to forego performing BSE behavior. Since we cannot be certain that the current participants were indeed unrealistically optimistic about their health (behaviors), further research is necessary to investigate the association of cues to action with unrealistic optimism and with less engagement in BSE practice, particularly in light of how the variables were operationalized in the present study.

An additional possible explanation for the negative association between cues to action and BSE performance may be found in the Indonesian culture. Modesty tends to be an important value for Indonesian women, and this trait could encourage the belief that the breast is an intimate, personal organ, not to be openly discussed. These cultural characteristics may bring about defensive and protective thoughts and a hesitation to consider performing BSE, since it is considered an uncomfortable procedure [40].

In line with a finding among the Saudi population [13], our data demonstrated that perceived severity and susceptibility to breast cancer did not predict BSE performance, and the mean scores for these variables did not differ between the BSE performers and the non-performers. There are several possible explanations of this finding. First, women might have insufficient knowledge about vulnerability to breast cancer, the severity of the disease, and how it influences the physical, psychological and social aspects of the patients and their family. Second, is the participants in the present study might interpret their susceptibility to suffering from breast cancer as the will of God. Previous studies indicated that such fatalistic beliefs negatively affect early detection behaviors in Muslim societies [41, 42]. The Indonesian population, is characterized by strong religious values, and our was drawn from a largely Muslim population. Future research should focus on establishing the underlying reasons for the absence of a relationship between BSE performance – or other health-related behaviors – and perceived severity and susceptibility.

The finding that perceived severity and perceived vulnerability were not correlated with BSE practice could also be explained by fear arousal theory. Rogers and Deckner [43] defined a fear appeal as a persuasive form of communication that aims to arouse fear so as to promote precautionary motivation and self-protective action. Further, Ruiter, Kessels, Peter and Kok [44] explained that fear arousal is an unpleasant emotional state, consisting of physiological, cognitive, affective, and behavioral responses triggered by the perceived threatening stimuli. The severity of and personal susceptibility to breast cancer are likely to be viewed as threats, and thus arouse fear. Moreover, if there is an additional assessment indicates that one is unable to effectively avert the risk, or that this aversion is not easily overcome, then this threat perception will result in ongoing fear arousal. Indeed, previous research indicated that perceived severity and susceptibility, both of which are components of perceived threat, were weak predictors of intention and risk-reducing behavior [45–47]. Thus, in order for these variables to cause enduring behavior change, risk communication must be combined with other behavioral change techniques such implementation intentions, self-efficacy training, or action instruction [44].

Among the socio-demographic variables examined, older age, well-educated and having family members with breast cancer were significantly associated with BSE practice. Our findings are consistent with those of Noroozi, Jomand, and Tahmasebi [15], who also reported that older women were more likely to perform BSE. It might be because they believe that the vulnerability of breast cancer is increasing with age. The results with regard to education indicated that BSE practice was also predicted by educational level, in which the odds to perform BSE were larger for women with higher education (i.e., high school and university graduates) compared to those with lower education (i.e. primary school). This finding is in line with those of studies in Turkey [14] and Singapore [48]. Finally, the association between family cancer history and greater BSE practice is in line with a study in a Saudi population, which showed that family history of cancer was significantly correlated with BSE performance [13]. One explanation of this relationship could be that women with relatives suffering from cancer understand the possible influence of genetic factors in the development of cancer, creating higher awareness of their own susceptibility. Marital status and history of breast affliction were not correlated with prior BSE performance in this sample.

Some limitations of the present study should be noted. First, we did not measure women’s current level of knowledge about breast cancer in general or BSE practice in particular. Future research should include such measurements, since breast-related knowledge – or health literacy in general – could be directly related to perceptions of susceptibility and severity. Further, the unexpected finding that cues to action decreased the likelihood of performing BSE, and the non-significant relationships between BSE practice and perceived susceptibility and severity, suggest that some of the women in our study may have had unrealistically optimistic perceptions of their own health and thus feel less urgency about performing BSE. Therefore, future studies should investigate the impact of optimistic bias on perceptions of breast cancer susceptibility and of the need to engage in screening behaviors. Lastly, the data were collected by means of self-report questionnaires, and asked women to report only whether they had ever performed BSE, not whether they were aware of (and thought they followed) the BSE guidelines. The difference between women’s self-reported knowledge and their actual knowledge, and the relationship between this difference and BSE practice, could be explored further in future studies as well.

## Conclusion

Our study provides some starting points and recommendations for health education. Given the significant association between perceived benefits of BSE and actual BSE practice, health promotion activities could focus on the benefits of BSE practice as an early detection strategy with respect to local context and culture. Expression of reluctance should be respected but can be addressed, such as by the use of female health educators. The result of self-efficacy was positively associated with BSE practice suggests that employing methods to increase women’s self-efficacy with regard to BSE, such as guided practice, or enactive mastery experiences, could improve women’s confidence and comfort level with regard to practicing BSE regularly. Further, our investigation of psychosocial variables among a large, representative sample of women makes generalization to the larger population possible.

## Data Availability

Data are available upon request, by sending an email to the corresponding author.

## Abbreviations

BSE: Breast Self-Examination
CBE: Clinical Breast Examination
CHBMS: Champion Health Belief Model Scale
HBM: Health Belief Model
I-CHBMS: Indonesian version of Champion Health Belief Model Scale
PHC: Primary Healthcare Center
PKK: Pembinaan Kesejahteraan Keluarga *(Women’s family welfare movement)*
